# Visual similarity in masking and priming: The critical role of task relevance

**DOI:** 10.2478/v10053-008-0026-z

**Published:** 2008-07-15

**Authors:** James T. Enns, Chris Oriet

**Affiliations:** 1 University of British Columbia; 2 University of Regina

**Keywords:** masking, priming, task-relevance, visual similarity, reentrant processing

## Abstract

Cognitive scientists use rapid image sequences to study both the emergence of conscious perception (visual masking) and the unconscious processes involved in response preparation (masked priming). The present study asked two questions: (1) Does image similarity influence masking and priming in the same way? (2) Are similarity effects in both tasks governed by the extent of feature overlap in the images or only by task-relevant features? Participants in Experiment 1 classified human faces using a single dimension even though the faces varied in three dimensions (emotion, race, sex). Abstract geometric shapes and colors were tested in the same way in Experiment 2. Results showed that similarity *reduced* the visibility of the target in the masking task and *increased* response speed in the priming task, pointing to a double-dissociation between the two tasks. Results also showed that only task-relevant (not objective) similarity influenced masking and priming, implying that both tasks are influenced from the beginning by intentions of the participant. These findings are interpreted within the framework of a reentrant theory of visual perception. They imply that intentions can influence object formation prior to the separation of vision for perception and vision for action.

## VISUAL SIMILARITY IN MASKING AND PRIMING: THE CRITICAL ROLE OF TASK RELEVANCE

*Visual backward masking* is the modern method of choice for studying the time-course of object perception in conscious experience (Enns & Di Lollo, 2000; Kim & Blake, 2005), and *masked forward priming* is the comparable tool for studying the unconscious processes involved in response preparation (Ansorge & Neumann, 2005; Klotz & Neumann, 1999). In a typical masking experiment, participants try to identify a briefly flashed image (called a *target*) that is followed closely in time and space by a second image (called a *mask*). If the mask resembles the target and appears very soon after it, the target can be difficult to see, sometimes to the point of being invisible. By studying the relationship between target visibility and the time that elapses before the mask appears, the time-course of object perception can be inferred (Bachmann & Allik, 1976; Enns, 2004).

A typical masked priming experiment involves the same stimulus sequence of two displays in rapid sequence. The only difference is that participants are now asked to respond as rapidly as possible to the identity of the mask. The briefly flashed display that appears just prior to this visible mask is now called the *prime*. A prime can have a strong influence on the speed with which the mask is identified, even when the prime is invisible to participants, speeding responses when it resembles the visible mask and slowing responses when it resembles a mask mapped to an alternate response. Examining the effectiveness of various primes allows researchers to infer the content of the representations used to identify the masks (Ansorge & Neumann, 2005; Beitmeyer, Öğmen, & Chen, 2004; Klotz & Neumann, 1999).

A fundamental question that remains unanswered is “To what extent do these two tasks rely on the same underlying mental processes?” That is, are the processes leading to conscious perception of the target the same ones that lead to priming in mask identification? The existing evidence is mixed on this question. On the one hand, some display factors seem to have the same direction of influence on both tasks, pointing to an underlying unity. For example, increasing the temporal interval between the first and second display increases both the visibility of the first display and the magnitude of the priming that occurs in identifying the second display (Vorberg, Mattler, Heinecke, Schmidt, & Schwarzbach, 2003). Increasing the luminance contrast of the first display has a similar effect on both tasks, improving the visibility of the first display and increasing the priming effect on the second display (Breitmeyer, Öğmen, Ramon, & Chen, 2005).

But the influences of other factors seem to disso-ciate the two tasks, pointing to separate neural systems responsible for the visibility of the first display and its priming effect on identifying the second display. For example, in many cases brief displays that cannot be discriminated above chance levels, and are therefore not even visible, still produce strong priming effects (Lleras & Enns, 2004; Vorberg et al, 2003). Some reports even claim that priming is strongest when the first display is never seen (Brietmeyer et al., 2005; Klapp & Hinkley, 2002; Schlaghecken & Eimer, 2002). Finally, the role played by perceptual similarity of the two displays appears to have opposite effects in the two tasks, with increased display similarity generally *reducing* first display visibility (see Breitmeyer, 1984, for a review) while at the same time increasing the priming effect for the second display (Ellis, Young, Flude, & Hay, 1987). However, to our knowledge the role of display similarity in the two tasks has never been compared directly in the same study.

Our first goal in this study was to examine the influence of image similarity in these two tasks, using precisely the same display conditions and the same participants in both tasks. Finding evidence that similarity plays an opposite role in the performance of these two tasks would then constitute strong evidence for a double dissociation, consistent with unique neural systems underlying these two tasks.

Our second goal was to determine whether the similarity effects in backward masking and masked priming were tied to physically defined features of the images or whether only task-relevant features participated in the similarity effects. This is an important question because the answer speaks to the levels of processing that are involved in both masking and priming. For instance, some theories propose that masking occurs at relatively early and low-levels of neural representation, prior to stages of visual processing during which the participant’s goals and intentions can have an influence on perception (Keysers & Perrett, 2002; Scheerer, 1973; Turvey, 1973). In the priming literature, some have also proposed that primes exert their influence independent of the goals and intentions of the participants (Jonides, 1981; Posner, 1980; Theeuwes, 1992, 1996; Winkielman, Berridge, & Wilbarger, 2005). If this is the case, for either masking or priming, then these tasks should be influenced by the physically defined similarity of the first and second display. That is, the effect of display similarity on masking and on priming should grow directly with the number and similarity of shared features in the two displays.

## A REENTRANT THEORY OF PERCEPTION

In contrast to the view that masking and priming are encapsulated from the intentions of the participant, our research has focused recently on the roles played by participants’ goals and their intended actions on the very earliest representations formed in the microgenesis of perception. Our ideas along these lines were first developed in studies of visual masking (Di Lollo, Enns, & Rensink, 2000; Enns & Di Lollo, 1997), but we have since applied them to studies of masked priming (Lleras & Enns, 2004; 2005; 2006), change blindness (Austen & Enns, 2000, 2003), the attentional blink (Di Lollo et al, 2005; Kawahara, Enns, & Di Lollo, 2003), the flash-lag illusion (Moore & Enns, 2004) and interrupted visual search (Lleras, Rensink, & Enns, 2005). In brief, visual perception is considered to be an iterative process whereby information is analyzed at several levels, most notably a higher level associated with object representations and a lower level associated with pre-categorical sensory input. Perceptual awareness is achieved once a “perceptual hypothesis” about a candidate object is created and confirmed by testing it against the current sensory input. Importantly, observers do not become aware of perceptual hypotheses that fail to be confirmed, which can happen when sensory information regarding the initial item is no longer present in the visual system, as is the case in visual backward masking.

According to this theory, the task of reporting the identity of the first of two images in a rapid sequence of displays will be influenced by somewhat different factors than the requirement to respond as rapidly as possible to the second of two images in the sequence. Consider first the case of a participant trying to identify the first image (i.e., a standard prime identification task). The participant must first form or activate a hypothesis about the image and then confirm that hypothesis by testing it against the available sensory evidence, before they are able to report on its identity. If the display changes before they have had the opportunity to confirm their initial hypothesis, there will be a mismatch between the hypothesis (based on the prime) and the new sensory information (the changed image). The system will have to be reset and a new hypothesis will be initiated, based on this new image. This is the account of the reentrant theory for successful backward masking of an image. Critically, because conscious awareness of an image is required as part of the task, a perceptual match must be established and this requires not only a feed-forward sweep of processing but also at least one feedback phase of processing.

Next, consider the case of a participant prepared to respond as rapidly as possible to the identity of the second image (i.e., a standard mask identification task). In this case, information regarding the various response alternatives can be sampled more or less continuously until enough evidence has accumulated to warrant committing to a response. There is no requirement that the sensory evidence must result in explicit perceptual awareness before a response can be made; only that there is sufficient sensory evidence to initiate one response rather than the other. Presentation of the prime will activate its associated response, whether conscious awareness of the prime follows or not (Cressman, Franks, Enns, & Chua, 2007; Lleras & Enns, 2006). If a second image maps to the same response, the evidence required for responding will accumulate to threshold faster than if the second image maps to a different response and the accumulation of evidence for the correct response must start over. The participant in a mask identification task will, of course, try to ignore the information entering the system from the first display, but to the extent that the first image is in the same location, and/or is difficult to discriminate from the second image in time, and/or shares visual features with masks assigned to the response classes, it will be difficult to disentangle the processing arising from the first image and forward response priming will ensue (Huber, Shiffrin, Lyle, & Ruys, 2001; Lleras & Enns, 2004; Weidemann, Huber, & Shiffrin, 2005).

As this brief summary makes clear, according to the reentrant theory of perception, for both kinds of tasks (prime and mask identification) information relevant to responding to either image is being sampled for a period of time that extends beyond the brief presentation of the image. Ordinarily, when perceiving dynamic events in natural settings, such temporal overlap in neural activity from discrete events helps the system to bridge brief gaps in input (Di Lollo, 1980) and to interpret distinct physical events in nearby locations as the same object moving or changing its appearance, a bias we refer to as *object-updating* (Enns, Lleras, & Moore, in press). In the artificial setting of the lab, however, where the participant is asked to respond selectively to the components of dynamic sequences, these processes favoring object continuity can lead to confusion. Moreover, this confusion is intensified when the task requires discriminating sensory evidence that arises from the prime versus a mask that is highly similar (as in a prime identification task). In the mask classification task, discriminating the source of the sensory evidence is less important than determining whether there is more of it in support of one response or another. Thus, confusion arises in mask classification when the sources of sensory evidence suggest conflicting responses; by the same token, facilitation results when both sources point to the same response.

According to the reentrant theory, both of these tasks can also be influenced by the intentions of the participant. If the participant is able to form a well-defined expectation of the target or class of target objects that are likely to appear prior to the onset of a display, then the process of hypothesis activation should take less time than if there is less certainty about the images that will be displayed (Di Lollo et al., 2000). Thus, for both prime and mask identification, performance should be strongly influenced by the degree to which the participant has been able to form a well-defined task template or filter for the anticipated display prior to its onset. By the same token, task-relevant features should be more likely than task-irrelevant features to influence performance in both tasks, especially if the participant can restrict processing to a narrow range of hypotheses.

This aspect of the reentrant theory is consistent with research from many other studies showing that perception is strongly influenced by expectations. For example, participants anticipating change in the identity of a face are faster to detect those changes than changes in emotional expression. Conversely, participants expecting changes in emotional expression are faster to detect those than changes in identity (Austen & Enns, 2003). Similarly, search for a target in a display that is periodically interrupted is not adversely affected by changes in target features that are irrelevant to the target detection response; changes to response-relevant features, on the other hand, slow down search significantly (Lleras, Rensink, & Enns, in press). Computational models of expectation effects have even been developed to account for the behavior of single cells in the striate cortex (Bridgeman, 1993).

To summarize, the identification of the first or the second of two images in a rapid sequence are both predicted by reentrant theory to be influenced by factors that bias the perception of a single object in a dynamic sequence and by factors that influence the range of possible perceptual hypotheses in a task. What is critically different in the requirements of the two tasks is that prime identification cannot occur before a successful match has been established between the feedback sweep of activity generated by an object hypothesis and the currently-available feed-forward sensory evidence (i.e., conscious awareness is a prerequisite for making a response). Mask identification, on the other hand, can occur without the need for a match, that is, it can proceed without the requirement of a feedback sweep of activity to fully confirm a particular hypothesis in the current sensory activity.

## OVERVIEW OF STUDY

If similarity effects in either visual masking or masked priming tasks are determined mainly by the goal-directed intentions of the participant, it would be strong evidence against the idea that these phenomena are complete at early levels of representation, that is, at levels encapsulated from the effects of goal-directed perception. In Experiment 1 we tested this idea by presenting pictures of human faces to participants in both a masking and a priming task. These were faces of many different individuals and they varied systematically in the emotions portrayed (either anger or happiness), in the race of the individuals (either Asian or Caucasian), and in their sex (either female or male). However, each participant classified the faces in both tasks using only one of these three dimensions (emotion, race, or sex). The results showed that similarity effects in masking were restricted to task-relevant features and that masks with similar task-relevant features *reduced* the visibility of the target face. The similarity effects in priming were also restricted to task-relevant features, but in this case these similar task-relevant features *increased* the speed with which the mask could be correctly classified.

The perception of human faces may be unique, either because of their biologically privileged status or because of a lifetime of acquired expertise. In Experiment 2 we therefore used a similar design, but tested the masking and priming of geometric shapes and colors to see if our findings generalized beyond faces. The results we obtained were substantially the same as in Experiment 1.

These experiments provide clear answers to our two questions. First, there is a double dissociation between masking and priming with regard to the influence of display similarity: similar masks are most effective in *reducing* target visibility in a masking task and similar primes are most effective in *increasing* the speed of responses to visible masks. This finding is consistent with unique neural systems underlying these two tasks. Second, it is task-relevant features and not the total number of shared features that govern the similarity effects in both masking and priming. This finding strongly suggests that the representations involved in these two tasks are influenced from the earliest stages by the goals of the participant.

## EXPERIMENT 1: FACES VARYING IN EMOTION, RACE, AND SEX

### Method

#### Participants

Forty-eight undergraduates from the University of British Columbia participated in a 1-hr session in return for partial course credit. Participants were assigned to one of three Relevant Feature conditions (emotion, race, sex). All participants reported normal or corrected-to-normal vision.

#### Displays and apparatus

 Displays were controlled by an eMac computer and presented centrally on a 17-inch CRT monitor at a viewing distance of approximately 50 cm (screen resolution 1024 x 768 pixels, 256 levels of gray, 89 Hz). There were a total of 16 different images of individual faces: 2 emotions (angry, happy) x 2 races (Asian, Caucasian) x 2 sexes (female, male) x 2 exemplars (person 1, person 2). Images were selected from the JACFEE set by Matsumoto and Ekman (1988). Images were 7.5 cm square (245 pixels per side), which corresponded to 8.6 degrees of visual angle per side. The background screen was an intermediate gray (50% intensity, 30 cd/m^2^) and the luminance of the faces ranged from a low of 10 cd/m^2^ (black hair regions) to a high of 90 cd/m^2^ (white skin regions). 

**Figure 1.**
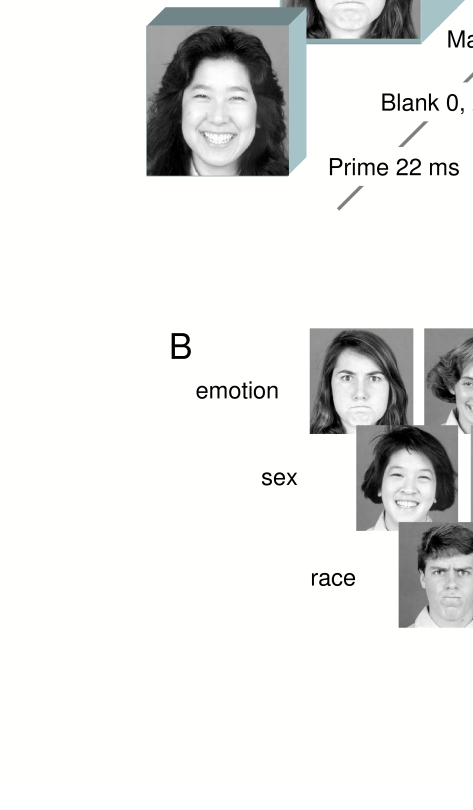
(**A**) Illustration of the display sequences in Experiment 1. In the mask classification task participants indicated as rapidly as possible either the emotion (angry, happy), the race (Asian, Caucasian) or the sex (female, male) of the mask face. In the prime classification task, participants made these same judgments of the prime face as accurately as possible. (**B**) Examples of the faces used in Experiment 1.

Each trial consisted of the following display sequence as shown in Figure 1: a prime face was presented for 22 ms, followed by a blank gray interval of 0, 22, or 45 ms, and then a mask face was presented for 504 ms. Response feedback was given for both tasks in the form of a plus sign (correct response), minus sign (incorrect response) or circle (no response) at the center of the screen, and remained on view for 1.5 s. This also served as the fixation point and warning symbol for the start of the next trial, which began 0.5 s after the feedback symbol was erased. Participants were given 2 s to make a response.

#### Procedure

Each participant first performed the speeded RT task of classifying the mask face according to the relevant feature they had been assigned (i.e., emotion, race, or sex), before performing the task of identifying the prime face according to the same feature. In the mask classification task, participants were told that ½ of the faces would be of each response type (i.e., angry or happy, Asian or Caucasian, female or male) but that they would be presented in random order. Participants were given printed and verbal instructions, before beginning a practice block of 10 trials. A testing session consisted of four blocks of 90 trials (360 trials in total). At the end of each block, a dialogue box on the screen indicated the error rate, and a warning message was presented if errors exceeded 5%. Participants were instructed to slow down on the next block if this warning message was presented. Response time (RT) was measured in milliseconds (ms).

In the prime classification task the display sequences and instructions were the same except that now the participant was asked to classify the relevant feature of the prime face. Here only accuracy was recorded as the dependent measure and participants were told to guess if they were uncertain about the identity of the prime face.

### Results

#### Speeded task classification

**Figure 2.**
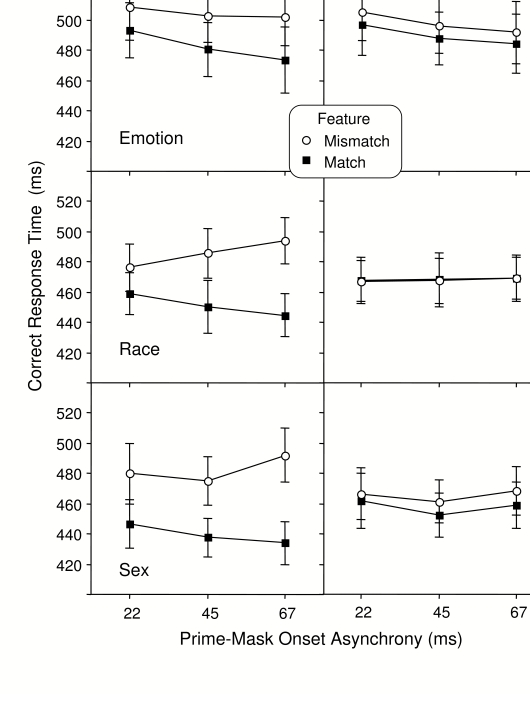
Mean correct RT in the mask face classification task. Feature match and feature mismatch refers to the relation between the prime and the mask faces. Relevant features are those used explicitly by the participant to classify the faces; irrelevant features are those that vary to the same degree but are not the basis for the classification. Error bar represents one standard error of the mean (SE).

Participants were very accurate overall (mean accuracy exceeded 95% in each group) and mean correct response time (RT) in milliseconds (ms) is shown in Figure 2. The left hand column of this figure shows RT when features of the prime and mask are matching or mismatching on the relevant features of the task; the right hand column shows RT when prime and mask are matching or mismatching on irrelevant features.

RT priming was influenced much more by the rele-vant than by the irrelevant features of the task. An analysis of variance (ANOVA) comparing RT for the three relevant features (left column in Figure 2) indicated that although there were no group differences in overall RT, *F*(2, 45) = 1.10, *p* =.35, *MSe* = 25392.60, matching features resulted in significantly shorter RT than mismatching features, *F*(1, 45) = 103.92, *p* =.001, *MSe* = 733.49, and this positive priming effect was larger for the features of race and sex than for emotion, *F*(2, 45) = 3.67, *p* =.04, *MSe* = 733.49. Moreover, the positive priming effect increased with prime-mask interval for all relevant features, *F*(2, 90) = 6.48, *p* =.01, *MSe* = 501.61, averaging 21 ms when the prime-mask onset interval was 22 ms and increasing to 46 ms when the interval was 67 ms in duration. This increase in positive priming with interval did not vary between groups of participants, *F*(4, 90) = 0.53, *p* =.71, *MSe* = 501.61.

An identical ANOVA comparing RT for the irrelevant features (right column in Figure 2) indicated a much smaller positive priming effect for the same sensory features of the faces when they were not related to the task of the participant, *F*(1, 45) = 8.88, *p* =.01, *MSe* = 210.26. This effect averaged only 5 ms and it did not vary significantly with the prime-mask interval, *F*(2, 90) = 0.07, *p* =.93, *MSe* = 248.13, or with participant group, *F*(4, 90) = 2.58, *p* =.09, *MSe* = 210.26. An ANOVA including feature relevance as a factor (comparing the left and right columns in Figure 2) indicated a significant 3-way interaction of Group x Relevance x Feature Matching, *F*(2, 45) = 4.64, *p* =.02, *MSe* = 381.02.

#### Prime classification accuracy

**Figure 3.**
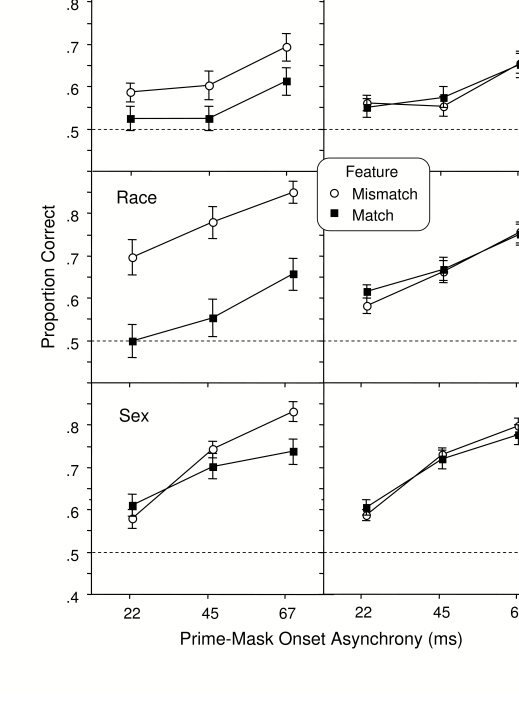
Mean proportion correct in the prime face classification task. Feature match and feature mismatch refers to the relation between the prime and the mask faces. Relevant features are those used explicitly by the participant to classify the faces; irrelevant features are those that vary to the same degree but are not the basis for the classification. Error bar represents one standard error of the mean (SE).

Figure 3 shows accuracy in the prime classification task separately for task relevant (left column) and irrelevant features (right column). The dashed line at an accuracy of .50 indicates the chance level of guessing in this two-alternative forced choice task.

The visibility of the prime face was influenced much more by the relations between the features of the prime and mask when the features were relevant to the discrimination being made. An ANOVA comparing accuracy for the three relevant features (left column in Figure 3) indicated that accuracy was generally higher for the sex group (mean = .70) than the race group (mea*n* = .67) or the emotion group (mean = .59), *F*(2, 45) = 9.80, *p* =.001, *MSe* = .032, and that accuracy increased along with prime-mask interval, *F*(2, 90) = 121.40, *p* =.001, *MSe* = .004. Prime accuracy was also much lower when the mask features matched those of the prime than when they mismatched, *F*(1, 45) = 18.34, *p* =.001, *MSe* = .042. This effect of feature similarity on prime visibility was greater for the race group (mean difference = .206) than for the emotion group (mean difference = .074) and the sex group (mean difference = .034) and it increased significantly with prime-mask interval only in the sex group, *F*(2, 30) = 10.47, *p* =.01, *MSe* = .003.

The same ANOVA comparing accuracy for the irrelevant features (right column in Figure 3) indicated that whether or not the features of the prime and mask matched one another had no influence on prime face visibility, *F*(1, 45) = 0.35, *p* =.56, *MSe* = .002.

An ANOVA including feature relevance as a factor (comparing the left and right columns in Figure 3) indicated a significant 3-way interaction of Group x Relevance x Feature Matching, *F*(2, 45) = 5.16, *p* =.01, *MSe* = .021.

#### Relations between tasks

The relations between performance on these two tasks was examined in several ways. First, the correlation between prime visibility (indexed by mean prime classification accuracy) and mask identification (indexed by mask classification RT) was not significant, *n* = 36, *r* = -.15, *p* =.37, suggesting that there was no direct link between prime visibility and the overall speed of mask processing. Yet, there were some factors that seemed to be related to performance in both tasks, including the *processing time given* exclusively to the prime (prime-mask interval), the extent to which the prime was *visible* (mean prime classification accuracy) and whether the prime and mask shared *task-relevant features* (mean difference between mismatching and matching relevant features). In this section we will consider each of these factors in turn.

Increasing prime processing time (the prime-mask interval) resulted in increases in prime visibility, but it had no direct effect on the speed of mask identification. Instead, longer prime processing reduced RT on feature-matching masks and lengthened RT on feature-mismatching masks. This leads to the hypothesis that longer prime processing time increases mask priming effects. However, this same variable did not increase the feature-relevant visi-bility effects in the prime detection task. As such, it appears that prime processing time influences both tasks (prime visibility and mask identification) but not in the same way.

One possibility worth exploring is that it is not the relationship between prime visibility and mask classification speed that is the important one, but rather the relationship between prime visibility and the net priming effect (RT difference between mismatching and matching trials). Examining this relationship, we find that increasing the processing time always increased the size of the priming effect for task relevant features. Considered on its own, this relationship suggests that prime visibility is directly related to response priming, speeding responses when there is a match and slowing it on a mismatch. But what this construal fails to explain is why the same magnitude of increase in processing time has no consequence when the matching-mismatching features are task-irrelevant (the right column of Figures 2 and 3).

Examining possible links between the tasks on the basis of *prime visibility* also seemed to have mixed effects, i.e., it depended on the factor used to alter prime visibility. On the one hand, increasing prime visibility by increasing the prime-mask interval led to larger priming effects, as already described, but increasing prime visibility by using a mask with mismatching relevant features led to a lengthening rather than to a shortening of the time needed to identify the mask. So, prime visibility is also not a factor that permits a unified understanding of prime visibility and mask identification speed. With regard to this issue, we note that several recent reports have claimed that primes that are processed exclusively at an unconscious level (i.e., that are effective as primes but invisible to the participant) result in response *inhibition* in a subsequent identification task involving similar features (Schlaghecken & Eimer, 2002, 2006). Conversely, primes that are perceived with awareness are thought to result in response *activation*. Directly relevant to this hypothesis, three conditions in the present experiment yielded prime accuracy levels that did not differ significantly from chance and therefore met a strict criterion for unconscious priming (feature matching conditions for emotion and race in Figure 3, left column). Yet, all three of these conditions resulted in strong positive priming in the mask identification task. As such, there was no support for prime visibility as a factor that unifies our understanding of these two tasks.

Task-relevant feature similarity was directly related to performance in each of these tasks, but the direction of influence was opposite in the two tasks. Similar relevant features in prime and mask *reduced* prime visibility (prime accuracy) whereas the same similar features *increased* the speed with which the mask could be identified. Task-relevant feature similarity is thus a factor that doubly dissociates the task of prime identification from that of mask identification.

### Discussion

This study clearly shows a double dissociation between the effects of image similarity on a visual masking task and a masked priming task. This occurred even though the only differences in the two tasks concerned the question posed to participants; identical image sequences were presented in each task. When participants tried to classify the face in the first display (masking task), similar faces in the second display were most effective in *reducing* target visibility. Conversely when participants tried to classify the face in the second display (priming task), similar faces in the first display were most effective in *increasing* the speed of classification. This is consistent with unique neural mechanisms in the two tasks.

The second important finding was that task-relevant features (not objective number of shared featues) governed the similarity effects in both tasks. This finding strongly suggests that the representations involved in these two tasks are influenced from the earliest stages by the goals of the participant.

We will have more to say about both of these findings in the General Discussion. However, it is first important to determine whether these results are peculiar to faces as images, or perhaps peculiar to backward masking involving overlapping patterns, or whether these results hold true more generally for other stimuli and other forms of backward masking. Faces may be treated as a special class of objects by the visual system for a number of reasons, including (1) their importance as meaningful signals of social-emotional-biological information, (2) the high degree of expertise that participants have acquired about faces over a lifetime of experience, or (3) the relational or configurational aspects of face processing. We also acknowledge that backward pattern masking also often gives rise to fundamentally different results than other forms of masking, such as simultaneous masking and metacontrast masking (Enns, 2004; Enns & Di Lollo, 2000).

In the next experiment we used a very similar experimental design, but instead of using faces as images, we used geometric shapes and colors as the features that could vary between images in the two displays. Also, instead of using pattern masking (in which the two images overlap one another in space) we used metacontrast masking, in which the contours of the first image fit snugly against, but do not touch, the contours of the second image.

## EXPERIMENT 2: GEOMETRIC SHAPES VARYING IN SHAPE AND COLOR

### Method

**Figure 4.**
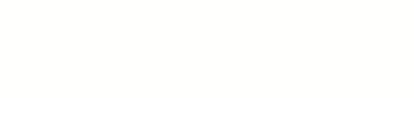
Illustration of the shapes and colors used in Experiment 2.

Thirty-six participants from the same pool as Experiment 1 were assigned to one of two Relevant Feature conditions (shape, color). Participants in the shape group first classified the second image as either a square or a diamond in the first half of the testing session (priming task) before classifying the first image as either a square or a diamond in the second half (masking task). Participants in the color group performed the same task using the same displays, but instead classified the images in each task as either blue or red. The prime and mask stimuli are shown in Figure 4.

There were a total of 4 different images used in first displays: 2 shapes (diamond, square) x 2 colors (blue, red). First display images were 0.9 cm per side (30 pixels). There were also 4 different second display images that were 1.8 cm per (60 pixels) side and contained a star-shaped hole that contained each of the first display images when they were overlaid. The color blue was composed of RGB values 0-0-100 and the red was composed of RGB values of 100-0-0. The background screen was white (RGB 100-100-100).

Trial sequences and procedures were otherwise identical to Experiment 1. In the mask classification task, participants were told that ½ of the shapes would be of each response type (i.e., diamond or square; blue or red) but that they would be presented in random order.

### Results

#### Speeded task classification

**Figure 5.**
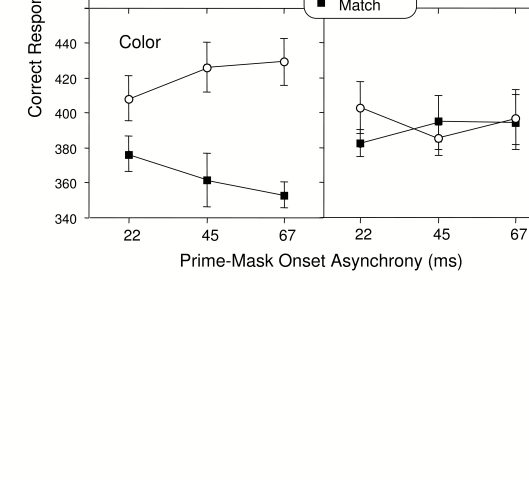
Mean correct RT in the mask classification task in Experiment 2. Error bar represents one standard error of the mean (SE).

Participants were very accurate in this experiment (mean accuracy exceeded 94% in each group) and mean correct RT is shown in Figure 5. The results were very similar to those for faces in Experiment 1, with a match between relevant features in the prime and mask resulting in positive RT priming, but not a match between irrelevant features.

ANOVA indicated that the color of the mask was responded to more quickly overall than the shape of the mask, and this effect approached significance *F*(1, 34) = 3.88, *p* =.06, *MSe* = 21694.70. With regard to priming, matching relevant features resulted in significantly shorter RT than mismatching relevant features, *F*(1, 34) = 37.96, *p* =.001, *MSe* = 4317.88, and this positive priming effect was larger for color than for shape, *F*(1, 34) = 8.58, *p* =.01, *MSe* = 4317.88. The prime-mask interval had no significant effect in this task with one exception that was marginally significant: when color was the relevant feature (lower left panel in Figure 4) RT increased with interval for mismatching colors and decreased with interval for matching colors, *F*(2, 68) = 2.97, *p* = .06, *MSe* = 1492.85. The ANOVA comparing RT for the irrelevant features (right column in Figure 4) indicated no other significant differences, all *Fs* < 1.13. An ANOVA including feature relevance as a factor (comparing the left and right columns in Figure 5) indicated a 2-way interaction of Relevance x Feature Matching, *F*(1, 34) = 17.17, *p* =.001, *MSe* = 2059.17.

#### Prime classification accuracy

**Figure 6.**
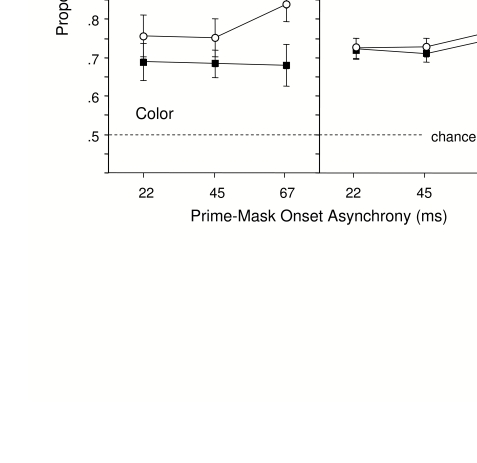
Mean proportion correct in the prime classification task in Experiment 2. Error bar represents one standard error of the mean (SE).

Figure 6 shows accuracy in the prime classification task for geometric shapes and colors. The dashed line at an accuracy of .50 indicates the chance level of guessing in the task. As was true for faces, the visibility of the prime was influenced by features it shared with the mask only when the features were relevant to the discrimination being made (left column in Figure 6). ANOVA indicated that accuracy was generally higher for color (mea*n* = .75) than for shape (mea*n* = .58), *F*(1, 34) = 42.87, p = .001, *MSe* = .029. Prime accuracy was lower when the mask features matched those of the prime than when they mismatched, *F*(1, 34) = 32.32, *p* =.001, *MSe* = .019. The prime mask interval did not have a significant influence, either as a main effect or in an interaction, *Fs* < 2.27, *ps* > .11.

The ANOVA comparing accuracy for the irrelevant features (right column in Figure 6) indicated that whether or not the features of the prime and mask matched one another had no influence on prime face visibility, *F*(1, 34) = 1.05, *p* =.32, *MSe* = .006. An ANOVA including feature relevance as a factor (comparing the left and right columns in Figure 5) indicated a significant 2-way interaction of Relevance x Feature Matching, *F*(1, 34) = 17.03, *p* = .001, *MSe* = .015.

#### Relations between tasks

The relations between the two tasks were examined in the same way as the previous experiment with faces. The overall correlation between prime accuracy and mask identification speed was again not significant, *n* = 24, *r* = -.282, *p* =.18, consistent with there being no simple relationship between prime visibility and the speed of mask processing.

Increasing the prime-mask interval in this experiment had no general effect on prime visibility or on the speed of mask identification. However, as in the previous experiment, longer prime processing time reduced RT on feature-matching masks and lengthened RT on feature-mismatching masks. Varying prime visibility also led to mixed effects: Increasing prime visibility by lengthening the prime-mask interval led to larger priming effects, but increasing it by using a mask with mismatching relevant features led to longer mean RT. With regard to unconscious priming, the three matching conditions for the shape group in the feature-relevant prime detection task did not differ significantly from chance (Figure 6, upper left panel) and yet these conditions led to strong positive priming in the mask identification task. And once again, task-relevant feature similarity had opposite directions of influence (i.e., led to a double dissociation) in the two tasks. The same relevant features *reduced* prime accuracy but *increased* the speed with which the mask could be identified.

### Discussion

These results with geometric shapes and colors (rather than faces), using a metacontrast masking procedure (rather than pattern masking), yielded essentially the same results with regard to our two main questions. First, image similarity reduced first-image visibility (masking task) and increased the speed of second-image classification (priming task). Second, the effect of similarity was significant only for image features that were relevant to the task being undertaken by the participant; equally large variations in the same features had no effect when those features were irrelevant to the goals of the participant.

## GENERAL DISCUSSION

These experiments are clear in providing evidence for: (1) A double dissociation in the effects of image similarity on a backward masking task and a masked priming task. Similar images were most effective in *reducing* target visibility in the masking task, as well as being most effective in *increasing* the speed of responses to visible masks. (2) Task-relevant similarity (not objective similarity) governed the similarity effects in both the masking and the priming task. The same physical features can therefore either influence masking and priming or not, depending on which features are relevant to the classification task the participant is actively engaged in. In this section we will discuss the theoretical implication of these two main findings in turn.

One general point that should be made first, however, is to acknowledge that there were masking effects in these experiments that were independent of the effects of the prime-mask similarity that were the focus of this study. That is, image similarity does not account for all the effects of prime visibility, nor presumably for all of the effects of priming on the task of rapidly classifying the mask image. There are other factors involved, including image contrast and the time between prime and mask. Therefore, bear in mind in the following discussion that we do not deny the importance of these factors. Rather, we will focus on the role that image similarity plays in addition to these other factors.

A second general question that should be addressed concerns whether the accuracy levels reported in the prime classification task of each experiment were contaminated by response bias effects (as opposed to being measures of what participants really experienced). Such a bias could come about, for example, if participants had a tendency to report the prime as “opposite to the visible mask” whenever they were uncertain about the prime’s identity (see Vorberg, Mattler, Heinecke, Schmidt, & Schwarzbach, 2004, for a method that is sometimes appropriate for ruling out effects of response bias, but that cannot be applied here because it requires averaging over the matching and mismatching conditions).

We believe there are several reasons why a response bias explanation is insufficient to account for all of the similarity effects in the prime visibility results. First, participants are told that the primes they are trying to classify consist equally of one type versus the other (e.g., equally angry versus happy in the emotion-relevant condition) and so there is no *a priori* reason we know of to select one bias (i.e., when uncertain, respond “opposite” to visible mask) over another (i.e., when uncertain, respond “same” as visible mask). At the same time, our theoretical perspective of reentrant processing provides plenty of motivation for predicting that perception will be biased by prime-mask similarity in this way. Second, there is a large and longstanding literature documenting that similar masks are more effective than dissimilar masks in reducing the visibility of a prime stimulus, even when response bias is not an issue because the measure of visibility is unrelated to the nature of the mask (Breitmeyer, 1984). Third, to the extent that there is a bias to respond “opposite” when uncertain in the present data, such an effect should reduce in size as the certainty of what is seen is increased (i.e., as the interval between prime and mask is increased). With increased visibility, any guessing strategy would be diminished. Yet the data show that the similarity effects on prime visibility, if anything, increase along with visibility; they are not decreased as predicted by this particular response bias interpretation.

### Unique neural systems involved in masking and priming

Ever since Fehrer and Raab’s (1962) report that simple RT to the onset of a metacontrast-masked target is unaffected by its visibility, vision researchers have been intrigued by the possibility that some visually guided actions can be accomplished without any accompanying awareness of the target shape that is responsible for the guided action. Since then, dissociations between conscious awareness and visually guided action have been studied in the literatures of visual geometric illusions (Carey, 2001), metacontrast masking of shape (Klotz & Neumann, 1999) and color (Schmidt, 2002), and the spatial location of targets in visually guided pointing (Chua & Enns, 2005; Goodale, Pelisson, & Prablanc, 1986) and grasping (Castiello, 1996; Ganel & Goodale, 2003). However, very little attention has been given to the role played by visual similarity in the two tasks that have been dissociated in these studies. The present study suggests that any theory of this dissociation must account for both the *opposite direction* that the influence of image similarity has on masking and priming tasks as well as for the finding that *only task-relevant features* have an influence on these similarity-based effects.

 From the perspective of the theoretical frameworks that are most commonly used to understand the dissociation in masking and priming, there is little reason to suppose that stimulus similarity should influence conscious perception and unconscious response priming in the same way (i.e., that only task relevant features play a role), and there is even less reason to suspect that these effects should be in opposite directions in the two tasks. For instance, within the *direct parameter specification* (DPS) theory of Neumann (1990), it is possible for participants to create a direct link between sensory information and the response parameters concerning when and how to respond. Once these are set up, they do not require mediation by conscious processes. A response is simply activated if the sensory activity contains features relevant to making a given response. Thus, within the DPS framework, where conscious and unconscious visual processes are considered separately, there is no expectation that the rules of visual similarity would be akin but opposite in their influence when two consciously perceived objects are similar. 

 Like DPS, the dual visual systems theory of Milner and Goodale (1995) is premised on *different neural systems* underlying unconscious, visually guided action and the conscious perception of objects and scenes. As such there is no built in expectation that similarity should influence each system in the same way. If anything, different rules governing similarity might be expected. This is because visually guided action is accomplished by the so-called dorsal visual stream, which extends from area V1 into the parietal lobe, whereas conscious perception resides in the so-called ventral stream that extends from area V1 into the temporal lobe. The dorsal stream is believed to be relatively color-blind and dominated by the fast-acting, magnocellular neural pathway that is sensitive to depth perception and motion. Most importantly for the guidance of actions, its spatial frame of reference is egocentric (from the perspective of the actor). The ventral stream, on the other hand, is believed to have relatively higher spatial acuity, to be color sensitive, and to represent objects in an allocentric frame of reference (from the perspective of the object). Thus, in this theory, there is also no reason to expect the rules of similarity for masking and priming should be so closely related to one another and yet opposite in their direction of influence. 

The reentrant theory of perception summarized in the introduction (Di Lollo et al, 2000; Enns et al, in press; Lleras & Enns, 2004) provides a different perspective on the dissociation observed for the effects of similarity. At the heart of this theory is the view that visual processes are inherently iterative because of the hierarchical nature of the receptive fields in the visually sensitive regions of the brain. As visual processing extends beyond area V1, receptive fields become simultaneously larger in their spatial scope and more complex in their feature specificity. Thus, in order to determine both “what” and “where” in any visual stream of processing (be it ventral or dorsal, for example) hypotheses must be activated and confirmed; one or more cycles of reentry is required to establish a stable representation (see Hochstein & Ahissar, 2002, for a review). From this perspective, it is expected that the biases of *object updating* (Enns et al, in press) should apply equally well to tasks performed by a visual subsystem that can result in conscious perception as well by a visual subsystem that can unconsciously guide actions based on parameters established prior to the appearance of a new object.

Note that this perspective suggests that the object updating processes underlying visual backward masking and masked priming are shared. This could come about because both the ventral and the dorsal visual streams begin by using the object representations that are instantiated through reentrant processing in cortical area V1. These representations can therefore be influenced by the goals and intentions of the participant, even though they are developed prior to the separation of further visual processing for the conscious ventral stream and the unconscious dorsal stream. This is admittedly a speculative hypothesis at this time, but we believe it accounts for the pattern of data revealed in the present study. Future studies will be required to determine if this hypothesis stands up to a priori tests.

The reentrant theory also provides a unique perspective on why the effects of similarity are opposite in their direction in the two tasks. Put simply, it is because “success” in the two tasks is rewarded by diametrically opposed task constraints. Consider first what it means to be correct in the masking task, where participants try to classify the first display in the face of a visual system inherently biased to update the earliest hypotheses activated by the first display with the features contained in the second display. “Success” in this task means one has been able to undo or “unbind” features that have been erroneously grouped together into one representation from the two displays. Not surprisingly, this should be easiest to do when the features in the two displays are most dissimilar, because they contain different feature values in shape, color, location, or even temporal characteristics.

“Success” in the priming task, on the other hand, involves responding rapidly to the second display. Thus, to the extent that the ubiquitous object updating biases of human vision favor an early preparation or initiation of the correct response to the second display, success in the task will be rewarded (given a head start) by similar prime images and punished (delayed) by dissimilar prime images. From the perspective of the reentrant theory, then, the opposite direction of influence of image similarity in masking and priming derives not from independent visual processing streams underlying the two tasks (although independent streams may indeed be the case), but rather from the requirements imposed by the different psychophysical tasks on visual representations that are biased to constantly update themselves in an effort to provide stable representations in the support of either conscious perception or accurate visually guided action. In a masking task, an inadvertently grouped rapid sequence of displays must be “unbound”; in a priming task, the same inadvertently “bound” rapid sequence of displays can influence perceptual-motor fluency (both positively if similar or negatively if dissimilar).

### Participants’ goals influence conscious and unconscious visual processes

The finding that similarity in the task-relevant features influenced masking and priming (but not similarity in the task-irrelevant features) strongly suggests that the representations involved in both of these tasks are influenced from the earliest stages by the goals of the participant. Let us consider the implications of this finding for each task in turn.

The finding that a participant’s goals influence visual-motor response priming implies that unconscious processes should not be equated with fixed or invariant processes, as is sometimes done. Instead, it points to the possibility that even unconscious visual processes are under the guidance and control of the high-level goals of the participant. When this point has been made previously in the context of tasks in which the displays can also be consciously experienced, as for example, in the contingent visual capture effects of Folk, Remington, & Johnston (1992), it has been less controversial than when similar points have been raised with respect to displays that are not consciously experienced (Ansorge & Neumann, 2005; Klotz & Neumann, 1999; McCormick, 1997; Schmidt, 2002). This is likely because, in the folk psychology of vision researchers, the concept of “unconscious” has been falsely associated with “zombie”-like processes rather than intelligent ones. However, just a moment’s reflection will reveal that even the most intelligent of processes relies heavily on a myriad of sub-processes that themselves never result in products of consciousness. Examples include the grammar of spoken language, shape constancy in visual perception, and reaching accurately for the handle of a coffee cup seen for the first time. So it may be time for researchers to abandon the intuitive, but unsupported links in their theories between unconscious and “dumb” (a term often used as shorthand for simple and invariant).

Indeed, when we look for other instances of intentions exerting an influence on unconscious visual processes, there is already a considerable and growing body of evidence pointing in this direction. For example, we have already mentioned work in our own lab showing that interrupted visual search (Lleras et al, in press) is influenced strongly by the expectations participants have about what features they will need to report in a psychophysical task. Although this study involves displays that ultimately result in conscious perceptions, the latency of the effects on manual RT are such that they occur *before* the time that participants are able to report on the contents of their perceptions.

Similar conclusions have been reached in the literature on the negative compatibility effect in masked priming (Lleras & Enns, 2006), where the short latency with which the prime influences motor processes (i.e., 100-200 ms, Verleger et al., 2004) is far below the time required for these same displays to result in visible images. The importance of task relevance has also been noted previously in the literature on response priming in metacontrast masking, where primes that are not visible influence responses to the visible mask, but only when their features correspond to the discriminations being made with regard to the visible mask (Ansorge & Neumann, 2005; Scharlau & Ansorge, 2003) or when the likelihood of a match between the prime and the mask features is high (Ansorge, 2004; Jaśkowski, Skalska, & Verleger, 2003).

 Turning to the role of task relevance in conscious perception, the finding that participants’ goals directly influence the effectiveness of a visual backward mask implies that the processes of masking are not accomplished in some invariant or pre-attentive stage of visual processing that passes its results on to a later “more intelligent” attentive or cognitive stage of processing. This has been the basis of quite a few general models of perception during the past few decades, including the influential feature integration theories of Neisser (1967) and Triesman (1988), and the two-stage models of rapid serial perception of Raymond, Shapiro, & Arnell (1992) and Chun & Potter (1995). But here too, there is already a growing body of evidence favoring a more interactionist view. For example, earlier we mentioned that participants anticipating change in the identity of a face were faster to detect identity changes than changes in emotional expression, and that participants with the opposite expectation were faster to detect changes in emotion (Austen & Enns, 2003). A recent report has extended this finding to the detection of two target faces in a rapid serial sequence of faces, with the result that similar targets are more difficult to detect only when their similarity is relevant to the features used to classify the faces (Sy & Giesbrecht, 2006). Stevanovski, Oriet, and Jolicoeur (2002) also reported a striking example of task relevance governing the influence of conscious perception. The perception of an ambiguous shape was impaired in that study by performing an action specific to one interpretation of the shape. When “<” was described as a left-pointing arrow, it was identified less accurately during a leftward than a rightward response. When the same “<” was described as a right-shining headlight, the opposite pattern of accuracy was observed. How participants intended to encode a shape therefore modulated their perception of it. 

### Conclusion

Understanding the relationship between conscious and unconscious processing in vision poses a considerable challenge for cognitive scientists. The present findings provide two important clues to this relationship. First, the finding that the conscious processes of object perception indexed in masking studies and the unconscious processes of action control tapped in priming studies are both strongly influenced by the intentions of the participant suggests that the early visual representations that guide both of these systems have much in common. The hypothesis we offer for further testing in this regard is that the reentrant processes we describe as *object updating* (Enns, Lleras & Moore, in press) are used to form the early representations that guide both of these systems.

Second, the finding of a double dissociation between masking and priming with regard to the influence of display similarity is consistent with the existence of at least partially unique neural systems underlying these two tasks (Milner & Goodale, 1995; Neumann, 1990) even though these systems may each make use of the same early visual representations. The hypothesis offered here for the double dissociation is that the purpose of conscious perception in a masking task (i.e., to see the first image without interference from the second image) is in direct conflict with the purpose of unconscious visually guided action in a priming task (i.e., to act rapidly on the information in the second image). Specifically, seeing the first image requires an “unbinding” of information that may already have been perceptually grouped when the rapid sequence was first processed. On the other hand, acting on the basis of the second image will be facilitated by earlier processing of related information, especially if that information is “bound” in early visual processing together with the second image. The challenge we set for future studies is therefore to test whether these speculative hypotheses withstand the scrutiny of future experimental data.
